# The spectral profile of cortical activation during a visuospatial mental rotation task and its correlation with working memory

**DOI:** 10.3389/fnins.2023.1134067

**Published:** 2023-03-16

**Authors:** Renata Figueiredo Anomal, Daniel Soares Brandão, Rafaela Faustino Lacerda de Souza, Sóstenes Silva de Oliveira, Silvia Beltrame Porto, Izabel Augusta Hazin Pires, Antonio Pereira

**Affiliations:** ^1^Morphology Department, Federal University of Rio Grande do Norte, Natal, Brazil; ^2^Brain Institute, Federal University of Rio Grande do Norte, Natal, Brazil; ^3^Department of Psychology, Federal University of Rio Grande do Norte, Natal, Brazil; ^4^Digital Metropolis Institute, Federal University of Rio Grande do Norte, Natal, Brazil; ^5^Laboratory of Signal Processing, Institute of Technology, Federal University of Pará, Belém, Brazil

**Keywords:** intelligence, alpha rhythm, mental rotation, working memory, EEG

## Abstract

**Introduction:**

The search for a cortical signature of intelligent behavior has been a longtime motivation in Neuroscience. One noticeable characteristic of intelligence is its association with visuospatial skills. This has led to a steady focus on the functional and structural characteristics of the frontoparietal network (FPN) of areas involved with higher cognition and spatial behavior in humans, including the question of whether intelligence is correlated with larger or smaller activity in this important cortical circuit. This question has broad significance, including speculations about the evolution of human cognition. One way to indirectly measure cortical activity with millisecond precision is to evaluate the event-related spectral perturbation (ERSP) of alpha power (alpha ERSP) during cognitive tasks. Mental rotation, or the ability to transform a mental representation of an object to accurately predict how the object would look from a different angle, is an important feature of everyday activities and has been shown in previous work by our group to be positively correlated with intelligence. In the present work, we evaluate whether alpha ERSP recorded over the parietal, frontal, temporal, and occipital regions of adolescents performing easy and difficult trials of the Shepard–Metzler’s mental rotation task, correlates or are predicted by intelligence measures of the Weschler’s intelligence scale.

**Methods:**

We used a database obtained from a previous study of intellectually gifted (*N* = 15) and average intelligence (*N* = 15) adolescents.

**Results:**

Our findings suggest that in challenging task conditions, there is a notable difference in the prominence of alpha event-related spectral perturbation (ERSP) activity between various cortical regions. Specifically, we found that alpha ERSP in the parietal region was less prominent relative to those in the frontal, temporal and occipital regions. Working memory scores predict alpha ERSP values in the frontal and parietal regions. In the frontal cortex, alpha ERSP of difficult trials was negatively correlated with working memory scores.

**Discussion:**

Thus, our results suggest that even though the FPN is task-relevant during mental rotation tasks, only the frontal alpha ERSP is correlated with working memory score in mental rotation tasks.

## 1. Introduction

The concept of intelligence is a timeless and fundamental aspect of human cognition, predating recorded history. It originated from observations of individuals attempting to solve practical, everyday problems (Hambrick et al., 2019). A key factor in successful problem-solving is the ability to create a spatial representation of the environment (National Research Council, 2006). In humans, there is a clear evolutionary connection between complex cognition and visual spatial abilities. These abilities are essential not only for navigation and route planning, but also for organizing the surrounding space. A recent study (Kedar et al., 2022) has demonstrated that early human groups were able to strategically position their hearths within paleolithic caves to avoid smoke suffocation. This further emphasizes the critical role of spatial intelligence in human survival and success.

The robust association between visuospatial skills and intelligence has many examples in the careers of notable physicists, mathematicians, inventors, and other professions of the science, technology, engineering, and mathematics (STEM) areas (National Research Council, 2006). In the educational context, several studies have shown that spatial ability not only correlates significantly with students’ interest and performance in STEM disciplines, even above mathematical and language skills, but also influences their future occupational choices (Stieff and Uttal, 2015). More interestingly, researchers have shown that spatial ability is a teachable ability (Stieff and Uttal, 2015) and enhanced spatial skills can help improve grades and retention rates of STEM students (Veurink and Sorby, 2019).

Among visuospatial reasoning abilities, mental rotation is a pervasive and regular feature of everyday activities (e.g., driving, reading maps, filling the dishwasher, building Lego sets), while also being particularly important for STEM activities (Wai et al., 2009; Sisman et al., 2021). Mental rotation is the ability to transform a mental representation of an object to accurately predict how the object would look from a different angle (Shepard and Metzler, 1971). The pioneer studies by Shepard and Metzler (1971) showed that response time for parity judgment of same figures in their classic mental rotation task increased with increasing angular disparity, a linear function often referred to as the angular disparity effect.

Performance on the Shepard-Metzler’s mental rotation task (SMT) involves flexibly switching between cognitive strategies, namely a motor simulation-based mental rotation strategy and a working memory-intensive analytic approach based on task difficulty (Gardony et al., 2017). Thus, when the angular difference between the two figures in the test is large (i.e., the task is more difficult), the subjects’ strategy shifts from motor simulation to working memory (Gardony et al., 2017). This finding was validated by our previous results showing that subjects with high intelligence quotient (IQ) perform better than average-IQ subjects in the SMT when the disparity angle of the figures increases (Anomal et al., 2020). Moreover, since subjects rely more on cognitive and visuospatial than motor strategies during difficult trials, we would expect to see increased neural activity in frontal and parietal areas (Gardony et al., 2017). These regions form the frontal-parietal network, which is characterized by the integration of frontal areas associated with higher cognition and parietal areas subserving spatial cognition. The frontal-parietal network, according to the parieto-frontal integration theory (PFIT), provides the structural substrate for the interaction of decision-making top-down signals and visual-spatial bottom-up inputs necessary to solve spatial tasks, Jung and Haier (2007).

Electrical oscillatory activity is a prominent characteristic of human electroencephalographic (EEG) recordings and are believed to improve neural communication and information processing in cortical networks (Buzsáki and Draguhn, 2004). Alpha band oscillations (8–12 Hz) have been linked to various cognitive processes, including attention and the maintenance of information in working memory (Klimesch, 2012), among other cognitive processes. One seminal discovery in the history of alpha oscillations is the “Berger effect,” named after its discoverer, which refers to the decrease in alpha amplitude due to neural desynchronization when the eyes are opened or cognitive demands increase (Doppelmayr et al., 2005). This phenomenon (the suppression of alpha oscillations by incoming visual information) has supported the idea that alpha oscillations are a passive cortical phenomenon, meaning that they are simply a byproduct of other neural processes and do not play an active role in information processing or other cognitive functions (Pfurtscheller et al., 1996; Pfurtscheller and Lopes da Silva, 1999). Another domain-general process associated with alpha desynchronization is “gating by inhibition” (Jensen and Mazaheri, 2010) or the conception that alpha oscillations control or reduce brain activity in specific regions, thus permitting the selective filtering of information. When alpha oscillations are suppressed in response to a task or stimulus, this release of control allows for a heightened level of activity and information processing to occur in other parts of the brain (Wang et al., 2019). A recent study demonstrated that alpha oscillations monitor memory storage in a content-specific manner, not just by keeping track of the number of items but also their level of complexity (Chen et al., 2022).

Energetic trade-offs play an important role in brain size evolution (Isler, 2013). As brains get larger, neural networks are topologically optimized for energy efficiency since longer axon pathways are comparatively less energy-efficient (wiring costs) (Avena-Koenigsberger et al., 2018). The Cortical Neural Efficiency Hypothesis (NEH) of cognitive and motor control proposes that the metabolic profile of brain function adapts to behavioral demands through a cost-efficient process (Nakata et al., 2010; Dunst et al., 2014). Studies that support the Cortical Neural Efficiency Hypothesis (NEH) often use neuroimaging techniques such as Positron Emission Tomography (PET), Single-Photon Emission Computed Tomography (SPECT), Functional Magnetic Resonance Imaging (fMRI), and Electroencephalography (EEG) to examine the relationship between the allocation of metabolic resources in cortical regions and measures of intelligence quotient (IQ) or physical expertise. While some research supports the Cortical Neural Efficiency Hypothesis (NEH), linking intelligence quotient (IQ) and physical proficiency with more focused and lower (more efficient) brain activation (Haier et al., 1992, 2003; Rypma et al., 2002; Neubauer and Fink, 2009), other studies challenge the validity of this hypothesis. Some studies have found that the NEH is only applicable when the task difficulty is moderate to low (Doppelmayr et al., 2005), while others have found that during a visuospatial task with football scenes, football players showed greater parietal cortical activity than controls (Del Percio et al., 2019). These findings suggest that the relationship between cognitive or motor abilities and metabolic resource allocation in the brain is complex and may depend on various factors, such as task difficulty and domain-specific expertise.

In the present work, we used EEG to investigate the neural correlates of the association between cortical activation and visuospatial skills. We performed event-related spectral perturbation (ERSP) analyses (Makeig, 1993) and looked at changes in event-related desynchronization (ERD) of the EEG alpha band during performance of the visuospatial SMT by subjects belonging to two groups: adolescents with average IQ or in the very superior (gifted) range. ERD reflects a decrease in spectral power from baseline values and has been associated with cortical excitatory processes (Pfurtscheller and Lopes da Silva, 1999). Our hypothesis is that activation of the PFN but not of other areas will be correlated with IQ measures and behavioral data, such as accuracy and response time.

## 2. Materials and methods

### 2 1. Participants

All procedures were approved by the Ethics Committee of the Federal University of Rio Grande do Norte (UFRN; CAAE: 50197415.9.0000.5537). All participants or their legal guardians signed an informed consent for participating in the study. The participants had no diagnostic of neurological dysfunction and had no uncorrected visual impairments.

We used data from a database obtained from a previous study in our laboratory (see Anomal et al., 2020). Participants were assigned to either one of two experimental groups (control and gifted) according to scores obtained with the Wechsler’s intelligence scales (WISC and WAIS). The total IQ score is composed of the following sub-scores: (1) verbal comprehension, (2) perceptual organization, (3) working memory, and (4) processing speed (Wechsler, 2003, 2008). Total IQ scores equal to and above 130 were considered “very superior,” between 120 and 129 “superior,” and between 80 and 119 “average” (Weiss, 2006). The gifted group (*N* = 15) was composed of adolescents (13–21 y.o.) with “very superior” total IQ scores. An age-matched control group (*N* = 15) had total IQ scores ranging from 80 to 128 (average and superior). Subjects in both groups were enrolled in the Gifted Program of the Digital Metropolis Institute of UFRN.^1^ Participants were 19 male and 11 female, 24 right-handed and 6 left-handed, and the total IQ of the control group ranged from 94 to 121, and 129 to 143 in the gifted group.

### 2.2. Experimental design

**FIGURE 1:**
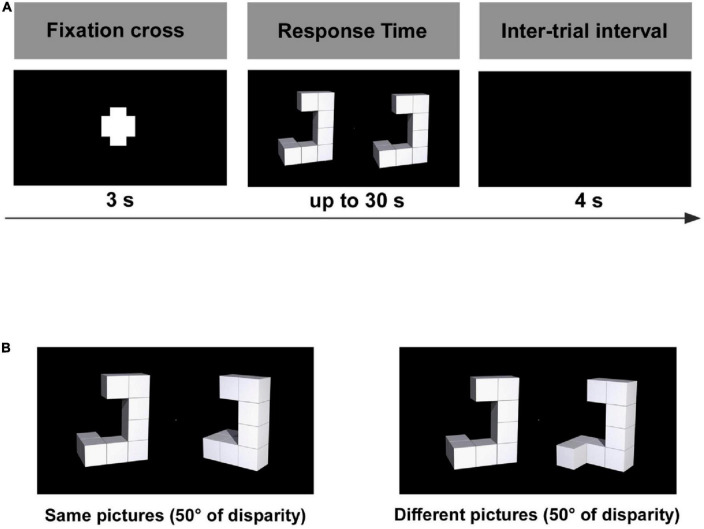
**(A)** Experimental design and **(B)** experimental conditions: same and different stimulus pairs (Anomal et al., 2020).

Participants performed a classic SMT (Shepard and Metzler, 1971), while their electroencephalographic signals were simultaneously recorded (see details below). The stimuli were presented on an LCD computer monitor (1,920 × 1,080 pixels) located 1.0 m in front of the participant. The experimental session contained 160 trials organized randomly. The experimental design shown in Figure 1 had the following sequence: (1) appearance of a fixation cross for 3 s, (2) display of stimulus for up to 30 s, and (3) inter-trial interval of 4 s (Neubauer et al., 2010; Anomal et al., 2020). When response times were longer than 30 s, the trial was halted and a new one was initiated by the software (PsychoPy, v1.90.d) (Peirce, 2007, 2009). The stimulus was composed of a pair of three-dimensional objects: the reference object on the left of the screen and the target located on the right (Ganis and Kievit, 2015; Anomal et al., 2020). Targets were rotated clockwise around the longitudinal axis in 50° increments from 0 to 150° (angle of disparity) and could be a mirror image of the reference image. In each trial, participants were asked to mentally rotate the target to determine whether they were the same or different (mirrored) from the reference. A total of 80 trials were presented for both conditions (same or different), with 20 trials for each angle disparity (0, 50, 100, and 150°).

Prior to the task, participants were briefed about the experiment’s design and purpose *via* a slide presentation and were instructed: (1) to perform a mental rotation to solve the test, (2) to press either the right or the left button of the mouse for the same or different condition, respectively, (3) to respond as fast as possible, and (3) to avoid committing errors.

### 2.3. Electroencephalographic recording and processing

The EEG was continuously recorded during task performance with a 1,000 Hz sampling rate from 64 Ag/AgCl cap-mounted electrodes organized according to the international 10–20 system (BrainAmp system, Brain Products). Eye movements were recorded by electrodes EOGz, EOG1, and EOG2, positioned at the glabella, and lateral to the left and right eye, respectively. The EEG recordings took place in a darkened room, with sound attenuation and temperature control. The electrical impedance of electrodes was kept under 25 k Ohms, and the signals were referenced to the electrode FCz. EEG data were analyzed with the EEGLab toolbox (Delorme and Makeig, 2004) running in Matlab (Mathworks, Inc., Natick, MA, USA). Channels were re-referenced to the average of all electrodes and band-pass filtered between 0.1 and 35 Hz. Ocular and muscular artifacts were removed through Independent Component Analysis. Electrodes with consistently poor signal quality were removed and reconstructed with interpolation using the PREP pipeline tool of the EEGLab toolbox.

The EEG was epoched to 500 ms before stimulus onset and 4,000 ms post-stimulus onset and submitted to a short-time Fourier Transform with a 250 ms Hanning window with frequency limits 0.5–40 Hz using the EEGLAB function “newtimef.” The resulting signal was then divided, point by point, by the average power of the full epoch. Corrected epochs were averaged by stimulus angle and subjected to division, also point by point, by the mean potency at baseline (–500 to 0 ms) to obtain the ERSP. Data from error trials and those with a signal amplitude above 100 μV were excluded from the analysis. We included only participants who performed at least 120 trials (75% of total trials).

Stimulus-related alpha (8–12 Hz) activity changes were computed by ERSP methods, which provide a 2-D representation of the mean change in spectral power (in dB) from baseline synchronized with the stimulus (Makeig et al., 2004). Differences in ERSP were investigated during the time interval which comprised the intervals of the rotation-related negativity (mental rotation interval) for the same pictures (963–1183 ms) observed in our previous work (Anomal et al., 2020). We did not analyze “different” pictures, or mirrored trials, because in these trials participants are supposed to use different strategies than mental rotation to solve the task, such as image flip (Hamm et al., 2004a,b). To perform our ERSP analysis, we employed a temporal window that was defined based on the event-related potential (ERP) interval exhibiting a stronger linear trend between stimulus angles. Specifically, we determined the onset and offset of the linear voltage changes related to orientation by identifying the time interval that exceeded the negative peak of the grand-averaged linear event-related potentials (ERPs) by 20%, following the approach described by Milivojevic et al. (2009). To analyze the ERPs of individual subjects for each of the four stimulus orientations (0, 50, 100, and 150°), we multiplied the ERP values by linear weight constants (–3, –1, 1, 3, respectively). We then scaled the resulting values for each orientation by the square root of the sum of the squares of the weights, as per standard practice.

### 2.4. Statistical analysis

Individual performance during the mental rotation task was expressed by both accuracy and response time measures (see Anomal et al., 2020). Regions of interest (ROIs) were defined based on the following electrodes: frontal (F1, F2, F3, F4, F5, F6, F7, and F8), parietal (P1, P2, P3, P4, P5, P6, P7, P8), temporal (T7, T8, TP9, TP10, TP7, TP8), and occipital (O1 and O2). Alpha ERSP was evaluated within the 8–12 Hz band. We investigated the effects of group (control and gifted), angle of disparity (0 and 150°), and region (parietal, frontal, temporal, and occipital) on alpha ERSP during RRN intervals. To examine these effects, we conducted a three-way mixed ANOVA (2 × 2 × 4), treating group as a between-subject factor and angle of disparity and region as within-subject factors. We selected the trials with 0 and 150° angles of disparity as easy and difficult trials, respectively, to assess the impact of angle of disparity on alpha ERSP values.

We used stepwise multiple linear regression to determine which factors predicted alpha ERSP, response time, and accuracy. For the alpha ERSP analysis, we calculated separate equations for each ROI (frontal, parietal, temporal, and occipital) and stimulus orientation (0 and 150° of disparity), using total IQ, working memory, perceptual organization, processing speed, and verbal comprehension as independent variables. The resulting equations were as follows:

(1) alpha ERSP (frontal ROI/0° of disparity) = B0 + B1 (total IQ) + B2 (working memory) + B3 (perceptual organization) + B4 (processing speed) + B5 (verbal comprehension) + u;

(2) alpha ERSP (parietal ROI/0° of disparity) = B0 + B1 (total IQ) + B2 (working memory) + B3 (perceptual organization) + B4 (processing speed) + B5 (verbal comprehension) + u;

(3) alpha ERSP (temporal ROI/0° of disparity) = B0 + B1 (total IQ) + B2 (working memory) + B3 (perceptual organization) + B4 (processing speed) + B5 (verbal comprehension) + u;

(4) alpha ERSP (occipital ROI/0° of disparity) = B0 + B1 (total IQ) + B2 (working memory) + B3 (perceptual organization) + B4 (processing speed) + B5 (verbal comprehension) + u;

(5) alpha ERSP (frontal ROI/150° of disparity) = B0 + B1 (total IQ) + B2 (working memory) + B3 (perceptual organization) + B4 (processing speed) + B5 (verbal comprehension) + u;

(6) alpha ERSP (parietal ROI/150° of disparity) = B0 + B1 (total IQ) + B2 (working memory) + B3 (perceptual organization) + B4 (processing speed) + B5 (verbal comprehension) + u;

(7) alpha ERSP (temporal ROI/150° of disparity) = B0 + B1 (total IQ) + B2 (working memory) + B3 (perceptual organization) + B4 (processing speed) + B5 (verbal comprehension) + u;

(8) alpha ERSP (occipital ROI/150° of disparity) = B0 + B1 (total IQ) + B2 (working memory) + B3 (perceptual organization) + B4 (processing speed) + B5 (verbal comprehension) + u.

To predict behavioral data, we created two equations using alpha ERSP from the four ROIs at 150° of disparity as independent variables and accuracy or response time as the dependent variable. The resulting equations were as follows:

(1) accuracy (150° of disparity) = B0 + B1 (alpha ERSP frontal) + B2 (alpha ERSP parietal) + B3 (alpha ERSP temporal) + B4 (alpha ERSP occipital) + u;

(2) response time (150° of disparity) = B0 + B1 (alpha ERSP frontal) + B2 (alpha ERSP parietal) + B3 (alpha ERSP temporal) + B4 (alpha ERSP occipital) + u.

In these equations, B0 represents the constant, and B1-B5 represent the coefficients of the independent variables, while u represents the standard error of the estimate.

We used Cook’s distance (Cook’s D) to identify outliers in the multiple linear regression and correlation analyses. ERSP values with a Cook’s D larger than 0.13 (4/n, considering n total = 30) were removed from the analysis.

We used the Shapiro-Wilk normality test to evaluate whether the data followed a normal (Gaussian) distribution. We calculated the mean squared error (MSE) and effect size (partial eta-squared: partial η2) for the ANOVAs. We used Sidak’s Method as a multiple comparison *post-hoc* test. For the correlation analysis, we used Pearson’s correlation coefficient or its non-parametric alternative, Spearman’s rank coefficient. Data were presented as mean ± SEM (standard error of the mean), and the criterion for significance was set at 0.05 for ANOVA analysis. When multiple analyses were performed, *p* was adjusted according to Bonferroni correction (0.05/n, *n* = number of analyses).

## 3. Results

### 3.1. Topographic map and regional activation during the mental rotation interval

A three-way repeated measures ANOVA was conducted to assess the effects of group (control, gifted), region (frontal, temporal, parietal, occipital), and angle of disparity (0, 150°) on alpha event-related spectral perturbations (ERSP). The analysis revealed a significant main effect of region [F (1, 28) = 17.522, MSE = 207.780, *p* < 0.001, partial η^2^ = 0.385], with the frontal region showing alpha ERSPs (–10.090 ± 2.232 μV) higher than in the parietal (–20.135 ± 2.061 μV; *p* < 0.001) and occipital regions (–17.716 ± 2.750 μV; *p* = 0.005), but not different from the temporal region (–10.676 ± 1.657 μV; *p* = 0.998). The alpha ERSP values recorded over the parietal ROI were lower than those recorded over the temporal (*p* < 0.001) and occipital ROIs (*p* = 0.795). In addition, there was a significant main effect of angle of disparity [F (1, 28) = 18.883, MSE = 207.780, *p* < 0.001, partial η^2^ = 0.403], with 150° showing higher alpha ERSP (–10.630 ± 2.052 μV) than 0° (–18.678 ± 2.259 μV; *p* < 0.001). Finally, there was no statistically significant interaction of alpha ERSP with group, region, and angle of disparity [F (1, 28) = 0.844, MSE = 207.780, *p* = 0.459, partial η^2^ = 0.029].

**FIGURE 2:**
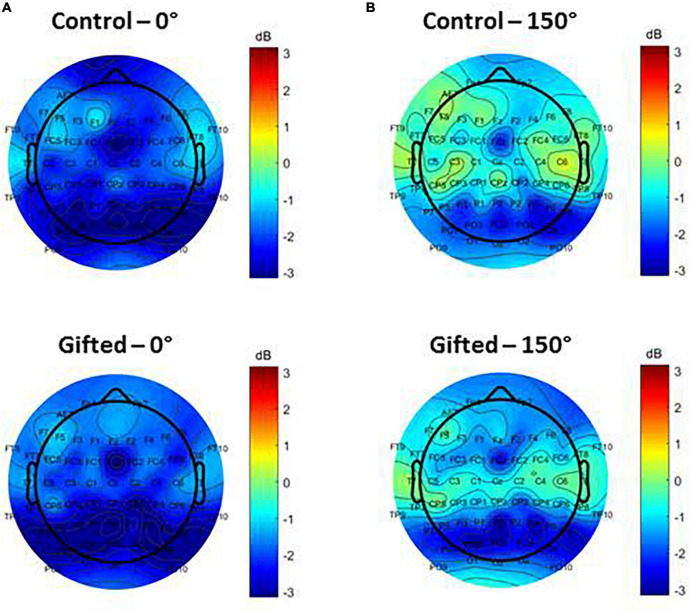
Electroencephalographic (EEG) scalp topography of alpha ERSP during the mental rotation interval (963–1183 ms). The maps are based on average alpha ERSP of the participants during trials with 0° **(A)** and 150° **(B)** of disparity between the template and target stimuli. Topographic maps of controls are on the top of the gifted group on the bottom. Values correspond to the mental rotation interval (defined by Anomal et al., 2020) and are color-coded according to ERSP’s amplitude in μV.

The topographic map of alpha ERSP for trials with 0 and 150° disparity did not indicate any significant differences between the control and gifted groups (Figure 2). However, for trials with 150° disparity, there was a noticeable trend toward more negative alpha ERSP values over the frontal regions of interest (ROIs) in gifted adolescents, compared to the control group (Figure 2).

### 3.2. Intelligence quotient and its sub-scores during the mental rotation interval (MRI)

After Bonferroni correction (*p* < 0.006, 0.05/8), multiple linear regression between alpha ERSP and intelligence scores for trials of 0° of disparity did not result in significant models for the frontal [F (1, 28) = 3.702, *p* = 0.019, R^2^ = 0.402], parietal [F (1, 28) = 3.054, *p* = 0.038, R^2^ = 0.357], temporal [F (1, 28) = 2.987, *p* = 0.041, R^2^ = 0.352], and occipital ROIs [F (1, 28) = 4.969, *p* = 0.016, R^2^ = 0.293].

**FIGURE 3:**
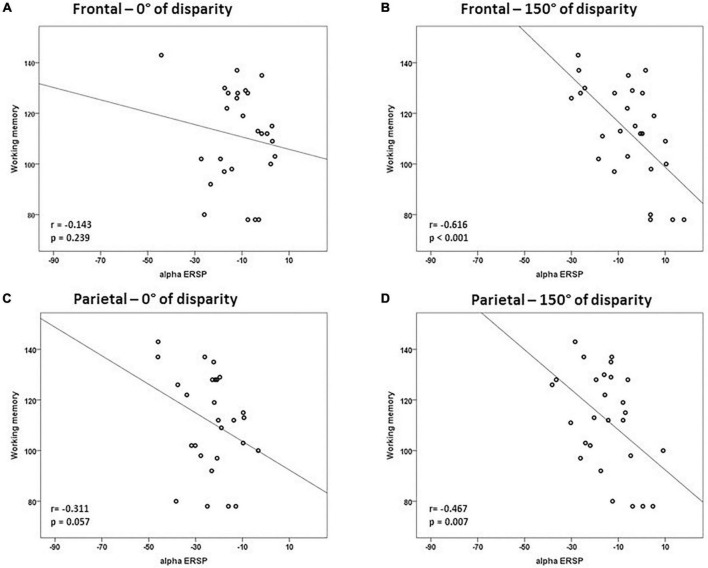
Correlations between intelligence scores and alpha event-related spectral perturbation (ERSP) in frontal and parietal regions of interest. There was a strong correlation between working memory scores and alpha ERSP values recorded over the frontal ROI during trials of 150° of disparity **(B)**. Correlations of easy trials of 0° of disparity are in panels **(A,C)**, and of difficult trials of 150° are in panels **(B,D)**. Values are in μV.

**FIGURE 4:**
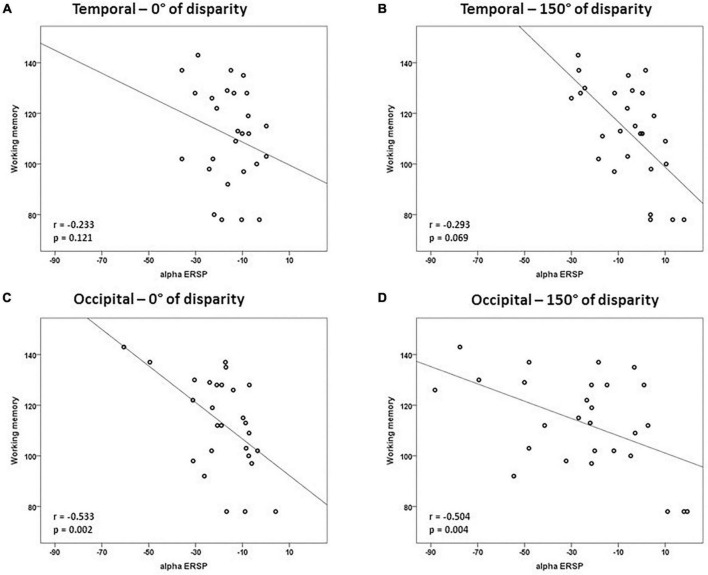
Correlations between intelligence scores and alpha ERSP in temporal and occipital ROIs. In the occipital ROI, there was a negative correlation between alpha ERSP values and working memory scores during trials with 0 and 150° of disparity between images. Correlations of easy trials of 0° of disparity are in panels **(A,C)**, and of difficult trials of 150° are in panels **(B,D)**. Values are in μV.

Considering that the results of the multiple linear regression for trials of 0° of angle disparity were not statistically significant, we tested the correlation between alpha ERSP and intelligence scores in easy and difficult trials. There was a negative correlation between alpha ERSP values recorded over the occipital ROI and working memory scores in trials of 0° of disparity (*r* = –0.533; *p* = 0.002; Bonferroni corrected) (Figure 3 and Table 1). For trials of 150° of disparity, alpha ERSP amplitude had a negative correlation with working memory scores in frontal (*r* = –0.616; *p* < 0.001) and occipital (*r* = –0.504; *p* = 0.004; Bonferroni corrected) ROIs (Figures 3, 4 and Table 1). No correlation was observed between the temporal ROI and intelligence scores for trials of 0 and 150° of disparity (Figure 4 and Table 1).

**TABLE 1:** Correlation coefficients between alpha event-related spectral perturbation (ERSP) values and intelligence sub-scores.

Alpha ERSP (0° of angle disparity)						
**ROIs**		**Intelligence quotient**	**Working memory**	**Perceptual organization**	**Speed processing**	**Verbal comprehension**
Frontal	R	-0.153	-0.143	-0.073	-0.263	-0.148
	*P*	0.224	0.239	0.360	0.093	0.231
Parietal	R	-0.308	-0.311	0.001	0.047	-0.259
	*P*	0.059	0.057	0.499	0.408	0.096
Temporal	R	-0.221	-0.233	0.136	0.141	-0.137
	*P*	0.134	0.121	0.249	0.241	0.248
Occipital	R	-0.328	-0.533	-0.278	-0.328	-0.333
	*P*	0.048	***0.002***	0.081	0.048	0.045
**Alpha ERSP (150° of angle disparity)**						
Frontal	R	-0.382	-0.616	-0.312	-0.315	-0.165
	*P*	0.025	**<*0.001***	0.056	0.055	0.206
Parietal	R	-0.089	-0.467	-0.074	-0.104	0.070
	*P*	0.330	0.007	0.358	0.303	0.364
Temporal	R	-0.221	-0.233	-0.295	0.141	-0.137
	*P*	0.132	0.121	0.067	0.241	0.248
Occipital	R	-0.212	-0.504	-0.165	-0.325	-0.074
	*P*	0.144	***0.004***	0.206	0.049	0.357

We considered the *post-hoc* correction for the above analysis (0.05/8, *p* < 0.006). Bolded italic values represent the statistically significant.

### 3.3. Behavioral data during the mental rotation interval

We conducted multiple linear regression analyses to investigate the relationship between behavioral data (response time and accuracy, Anomal et al., 2020) and alpha ERSP values recorded over the parietal, frontal, temporal, and occipital ROIs during difficult trials of 150° of disparity (Bonferroni correction *p* < 0.025, 0.05/2). However, the results did not yield a statistically significant model for alpha ERSP and accuracy [F (1, 28) = 1.405, *p* = 0.256, R^2^ = 0.149] or for alpha ERSP and response time [F (1, 28) = 1.364, *p* = 0.280, R^2^ = 0.206]. Considering the above results and the mentioned correlation between alpha ERSP and working memory in trials of 150° of angle disparity, we verified if there was a correlation between behavioral data and alpha ERSP. Table 2 shows that there were no significant correlations between alpha ERSP values and behavioral data during trials of 150° of disparity. While these results may seem counterintuitive, they suggest that alpha ERSPs may not be directly related to behavioral performance during difficult trials of the SMT.

**TABLE 2:** Correlation coefficients between alpha event-related spectral perturbation (ERSP) values and behavioral data (150° of angle disparity).

ROIs		Response time	Accuracy
Frontal	R	–0.016	–0.220
	*P*	0.468	0.130
Parietal	R	0.041	–0.313
	*P*	0.421	0.052
Temporal	R	–0.091	–0.100
	*P*	0.329	0.306
Occipital	R	–0.234	–0.231
	*P*	0.125	0.119

We considered the post-hoc correction for the above analysis (0.05/2, *p* < 0.025).

## 4. Discussion

There has been a longtime quest for the biological underpinnings of human intelligence, including its genetic basis and whether there are any differences between the brains of intellectually gifted and neurotypical individuals. This pursuit received a good amount of public attention in the XX century after the publication of a series of postmortem studies of Albert Einstein’s brain. Some findings from those studies included a higher ratio of glial to neuron cells and relatively enlarged parietal lobes (Diamond et al., 1985; Witelson et al., 1999). In a previous ERP study (Anomal et al., 2020), we showed that intellectually gifted adolescents not only outperform control subjects in a visuospatial task, but the profile of electrical activity in the brain of those two groups is also different, especially in the FPN. In the present work, we continue our search for the functional cortical substrates underlying superior intelligence and its relationship to visuospatial cognition. During difficult trials, our findings suggest that working memory scores have a predictive effect on alpha ERSP amplitude in both the frontal and parietal regions. In addition, we observed a negative correlation between working memory scores and ERSPs specifically in the frontal region. Our study yielded intriguing results, indicating a negative correlation between alpha ERSP values and working memory scores in the occipital region, regardless of task difficulty. Additionally, we found that the parietal region demonstrated significantly greater activation compared to the frontal, temporal, and occipital cortices. Interestingly, the frontal region exhibited lower activation than the parietal and occipital regions.

Our study employed visuospatial skill as a proxy for intelligence measures, a methodology supported by the close relationship between visuospatial skill and intelligence measures. Research has shown that there is a positive correlation between visuospatial abilities and overall intelligence, and that people with higher IQ scores tend to perform better on tests of visuospatial skills. Additionally, visuospatial skills are thought to play a role in the development of other aspects of intelligence, such as mathematical and scientific reasoning.

We demonstrated that in the FPN network, the parietal region is more active than the frontal, temporal and occipital regions during the mental rotation interval, as indicated by ERSP following stimulus appearance. Thus, we suggest that alpha power is up-modulated during task performance in these regions involved in the processing of task-irrelevant or distracting information, as an attentional suppression mechanism (Foxe and Snyder, 2011; Fiebelkorn and Kastner, 2019).

### 4.1. Intelligence scores and alpha ERSP

There has been conflicting evidence regarding the relationship between alpha ERSP and intelligence scores. Early studies indicated that during the execution of cognitive tasks, intelligence scores correlate positively with alpha ERSP (Grabner et al., 2003, 2004). Subsequent findings suggested that the positive correlation between intelligence scores and alpha ERSP depended on several factors, such as sex, task type, and fluid vs. crystalized intelligence scores (Neubauer et al., 1995, 1999, 2002, 2004; Grabner et al., 2003, 2004; Neubauer and Fink, 2003). Our findings indicate that alpha ERSP in the frontal cortex is negatively correlated with working memory scores in difficult trials of the SMT. More recent studies have suggested that alpha ERSP correlates negatively with intelligence scores and task performance (Klimesch et al., 1993, 1997; Doppelmayr et al., 2005). In mental rotation, task difficulty is represented by progressive increments of angle disparity between target and reference images and is reflected by increased response time (Shepard and Metzler, 1971) and higher RRN (Anomal et al., 2020). Using functional magnetic resonance imaging (fMRI), Lipp et al. (2012) showed augmented cortical activation with increased angles of disparity, in individuals with higher intelligence scores in the right frontal and inferior parietal cortex during a visuospatial task variant of the standard Posner task (Lipp et al., 2012). In accordance with our current findings, other studies have shown that lower and widespread alpha ERSP is more associated with complex than easy tasks (Dujardin et al., 1995; Wilson et al., 1999; Stipacek et al., 2003; Doppelmayr et al., 2005).

Our results of high frontal activation in adolescents with higher working memory scores are at odds with the NEH of intelligence (Haier et al., 1992; Haier et al., 2003), which suggests lower glucose usage in the brain of more intelligent individuals. It has been proposed that the interaction between the frontal and parietal cortex is metabolically costly for the brain (Bullmore and Sporns, 2012), because it involves coordinated activity between distant cortical regions and associated increased metabolic wiring costs. Hara et al. (2014) showed that scores in a delayed non-matching-to-sample task performed by monkeys is inversely correlated with the amount of malformed, energetically inefficient mitochondria in presynaptic boutons in the dorsolateral prefrontal cortex (Hara et al., 2014). This result and others suggest that the neuro-metabolism in prefrontal areas associated with higher cognition increases with task demands and can result in cognitive fatigue due to the necessity of recycling potentially toxic substances accumulated during cognitive control exertion (Wiehler et al., 2022). Thus, though gifted and average intelligence subjects display different oscillatory patterns in the alpha range, this may depend on moderating variables such as task difficulty, task type, sex, and brain area under investigation (Euler and Anna-Lena, 2021).

Further, according to our results, adolescents with a higher percentage of correct responses during the task, had low ERSP values in the frontal cortex, though they were not faster than their counterparts (Anomal et al., 2020). This is consistent with Yoon and Mann (2017) findings that students who spent more time on a spatial test tended to score higher than those who did not (Yoon and Mann, 2017). These results suggest that gifted students may be cautious or more perfectionists, taking longer to respond.

### 4.2. Working memory and the mental rotation task

In our study, during difficult trials, alpha ERSP was negatively correlated with working memory scores, but not with total IQ. In the Shephard-Metzler’s task, the underlying brain processing steps involve perceptual encoding, identification and discrimination of the objects, identification of their orientation, mental rotation, judgment of parity, response selection, and response execution (Heil, 2002; Heil and Rolke, 2002). We propose that working memory should be of particular importance during task performance, since the mental representation of the target object must be held online in working memory while being rotated and compared to the reference object (Hyun and Luck, 2007; Prime and Jolicoeur, 2010).

Our findings also show that working memory scores correlate negatively with alpha ERSP in the occipital cortex during both easy and difficult trials. The role of this region in working memory processing has been investigated since fMRI studies demonstrated that early visual areas can retain specific information about visual features held in working memory (Ester et al., 2009; Harrison and Tong, 2009), and that transcranial magnetic stimulation (TMS) over the occipital cortex reduces visual working memory consolidation (van de Ven et al., 2012). Another study of anodal direct current stimulation of the visual cortex, which increases neuronal excitability, shows improved visual working memory consolidation in a standard change detection task (Makovski and Lavidor, 2014).

We also observed that in difficult trials, alpha ERSP recorded in the parietal and frontal cortex is closely associated with working memory scores. This involvement of the parietal and prefrontal cortex in the retention of visual working memory information is already well-established (Miller et al., 1996; Todd and Marois, 2004). In a previous work, we had already shown that working memory scores are correlated with ERP amplitude in both the FPN during the rotation-related negativity interval of the SMT (Anomal et al., 2020). Other studies have shown that the communication between frontal and posterior regions during a working memory task is modulated according to the cognitive demands required for a successful performance (Fernández et al., 2021).

## 5. Conclusion

Our findings reveal a heightened activation of the parietal cortex in comparison to other regions, further emphasizing the significance of the Frontoparietal Network (FPN) in the successful completion of spatial mental rotation tasks (SMT). The correlation between working memory and alpha event-related spectral perturbation (ERSP) occurred in the frontal cortex and highlights the vital role of this region in working memory, which is a crucial aspect of SMT. Additionally, the link between occipital cortex activity and working memory scores supports our hypothesis that this region plays an important role in supporting working memory tasks.

Initially, it was believed that low cortical activation in highly intelligent individuals indicated a more economical use of brain glucose and efficient neural functioning. However, our findings also demonstrate that alpha ERSP amplitude is influenced by task difficulty.

One surprising outcome of our study was the correlation between alpha ERSP and working memory (WM), but not IQ. It is important to keep in mind, however, that although previous research has indicated a positive relationship between WM capacity and IQ, WM is only one of the many factors that contribute to a person’s intellectual ability (Engle, 2002). Other factors, such as motivation, education, and life history, can also have a significant impact on an individual’s IQ (Engle, 2002).

The correlation between mental rotation and intelligence has significant implications for STEM fields and careers, given their reliance on visuospatial ability, making it crucial to understand the relationship between the two. To enhance the development of visuospatial intelligence, we suggest that educational curricula should place more emphasis on activities relying on visuospatial ability, including mental rotation tasks.

However, a limitation of our study is the relatively small sample size, which precluded us from examining potential gender differences in brain activation patterns during visuospatial tasks. Previous research has shown that, on average, males generally score higher than females on measures of spatial ability, including visuospatial tasks (Linn and Petersen, 1985). In future studies, it would be interesting to increase the sample of both males and females and examine whether any gender differences exist in brain activation patterns during visuospatial tasks. Additionally, it would be interesting to explore the potential influence of cultural and social factors on these differences and disentangle their relative weight.

## Data availability statement

The original contributions presented in this study are included in the article/supplementary material, further inquiries can be directed to the corresponding author.

## Ethics statement

The studies involving human participants were reviewed and approved by Ethics Committee of the Federal University of Rio Grande do Norte. Written informed consent to participate in this study was provided by the participants’ legal guardian/next of kin.

## Author contributions

AP conceived the project. RA, SS, SP, and IH performed the experiments. AP, RA, RS, DB, and SS analyzed the data. AP and RA wrote the manuscript. All authors contributed to read the manuscript and approved the submitted version.

## Footnotes

1
www.metropoledigital.ufrn.br
